# Dissecting the abilities of murine Siglecs to interact with gangliosides

**DOI:** 10.1016/j.jbc.2024.107482

**Published:** 2024-06-17

**Authors:** Edward N. Schmidt, Xue Yan Guo, Duong T. Bui, Jaesoo Jung, John S. Klassen, Matthew S. Macauley

**Affiliations:** 1 Department of Chemistry, University of Alberta, Edmonton, Alberta, Canada; 2 Neuroscience and Mental Health Institute, University of Alberta, Edmonton, Alberta, Canada; 3 Department of Medical Microbiology and Immunology, University of Alberta, Edmonton, Alberta, Canada

**Keywords:** Siglec, ganglioside, glycolipid, liposome, mouse, flow cytometry, mass spectrometry

## Abstract

Siglecs are cell surface receptors whose functions are tied to the binding of their sialoglycan ligands. Recently, we developed an optimized liposome formulation and used it to investigate the binding of human Siglecs (hSiglec) against a panel of gangliosides. Animal models, more specifically murine models, are used to understand human biology; however, species-specific differences can complicate the interpretation of the results. Herein, we used our optimized liposome formulation to dissect the interactions between murine Siglecs (mSiglecs) and gangliosides to assess the appropriateness of mSiglecs as a proxy to better understand the biological roles of hSiglec–ganglioside interactions. Using our optimized liposome formulation, we found that ganglioside binding is generally conserved between mice and humans with mSiglec-1, -E, -F, and -15 binding multiple gangliosides like their human counterparts. However, in contrast to the hSiglecs, we observed little to no binding between the mSiglecs and ganglioside GM1a. Detailed analysis of mSiglec-1 interacting with GM1a and its structural isomer, GM1b, suggests that mSiglec-1 preferentially binds α2-3–linked sialic acids presented from the terminal galactose residue. The ability of mSiglecs to interact or not interact with gangliosides, particularly GM1a, has implications for using mice to study neurodegenerative diseases, infections, and cancer, where interactions between Siglecs and glycolipids have been proposed to modulate these human diseases.

Mice are among the most commonly used organisms for studying human physiological and pathophysiological processes (1). However, challenges arise in using mice as a model organism when proteins involved in processes and pathways differ substantially between mice and humans. The sialic acid–binding immunoglobulin-type lectins (Siglec) family of immunomodulatory receptors are a good example of proteins that are divergent between the two species (2, 3). Siglecs are cell surface receptors expressed by immune cells whose functions are regulated by their sialoglycan ligands (4, 5, 6). These functions include regulation of immune cell signaling, internalizing extracellular cargo, and cell adhesion (7). Siglec family members differ with respect to their specificity towards their sialic acid–containing glycoproteins and glycolipid ligands (8). Given that the immunomodulatory roles of Siglecs are directly tied to their ability to bind their ligands, it motivates a better understanding of their ligands, particularly as it relates to functional analogs between mice and humans.

Across mammals, Siglecs share characteristics such as the glycan-binding N-terminal V-set domain, at least one IgG-like C2 domain, and a single pass transmembrane segment (9, 10). Within the V-set domain, Siglecs have a conserved arginine residue that is essential for binding sialylated glycans (11). The cytoplasmic tails of Siglecs differ between family members, with the majority containing an immunoinhibitory motif that can antagonize immune cell signaling (10). Importantly, in humans, there are 15 Siglecs, while in mice, there are only nine Siglecs (8). Siglec-1, -2, -4, and -15 are well conserved between mice and humans with respect to their expression pattern and primary sequence similarity, but the rest are significantly divergent and classified as the CD33-related Siglecs (8). Among the CD33-related subfamily, there are orthologs, which have resulted from new genes evolving from a common ancestral gene and paralogs that result from a gene duplication event (12). For example, Siglec-7 and -9 are orthologs of Siglec-E, Siglec-8 is a paralog of Siglec-F, Siglec-10 is the ortholog of Siglec-G, and the rest are specific to their respective species (13).

Siglec ligands can be sialylated glycolipids (14, 15, 16, 17), which are primarily gangliosides on mature mammalian cells. The ganglioside content of a cell depends on the type of a cell. Gangliosides are most abundant in nervous tissues and are typically found in a range between 0.1 and 5 mol% of the total lipids in a cell (18, 19, 20, 21). Gangliosides are a family of glycolipids that share the carbohydrate backbone β-Gal*p*-(1 → 3)-β-Gal*p*NAc-(1 → 4)-β-Gal*p*-(1 → 4)-β-Glc*p*, which is linked to ceramide through the C1 hydroxyl of the glucose residue (22). Sialic acid can be linked to each of the monosaccharide units, except for the glucose (23). Siglec–ganglioside interactions were first investigated using ‘out-of-bilayer’ assays such as cell adhesion assays (24), ELISA (14, 25), or glycan microarrays (26, 27, 28). However, recent studies of Siglec–ganglioside interactions have used liposomes that enable the glycolipid to be embedded in a lipid bilayer (15, 29, 30). In particular, our recent work profiled the entire human Siglec family against a panel of gangliosides embedded in an optimized liposomal formulation, which revealed many new interactions (15). This work highlighted that the oligosaccharide portion of a ganglioside can be presented in a unique way from a lipid bilayer that can influence Siglec–glycolipid interactions. How bilayer oligosaccharide presentation affects murine Siglec–ganglioside interactions is largely unknown.

Motivated to understand the differences between the ability of Siglecs from mice and humans to recognize gangliosides, we used gangliosides presented from liposomes and a traditional ELISA approach to interrogate the murine family of Siglecs. Several key differences between glycolipid-binding profiles were observed between Siglecs from mice and humans. Although the murine Siglec–ganglioside binding profiles observed between the ELISA and the liposome assay largely agreed, unexpectedly, the ganglioside GM1a was not found to be a ligand for murine Siglecs (mSiglecs) when presented from a liposome. This may have implications for studying Siglecs in murine models, such as the ability of Siglec-1 to interact with gangliosides in viruses (31, 32, 33).

## Results

### Investigating mSiglec–ganglioside binding in a bilayer

**Figure 1:**
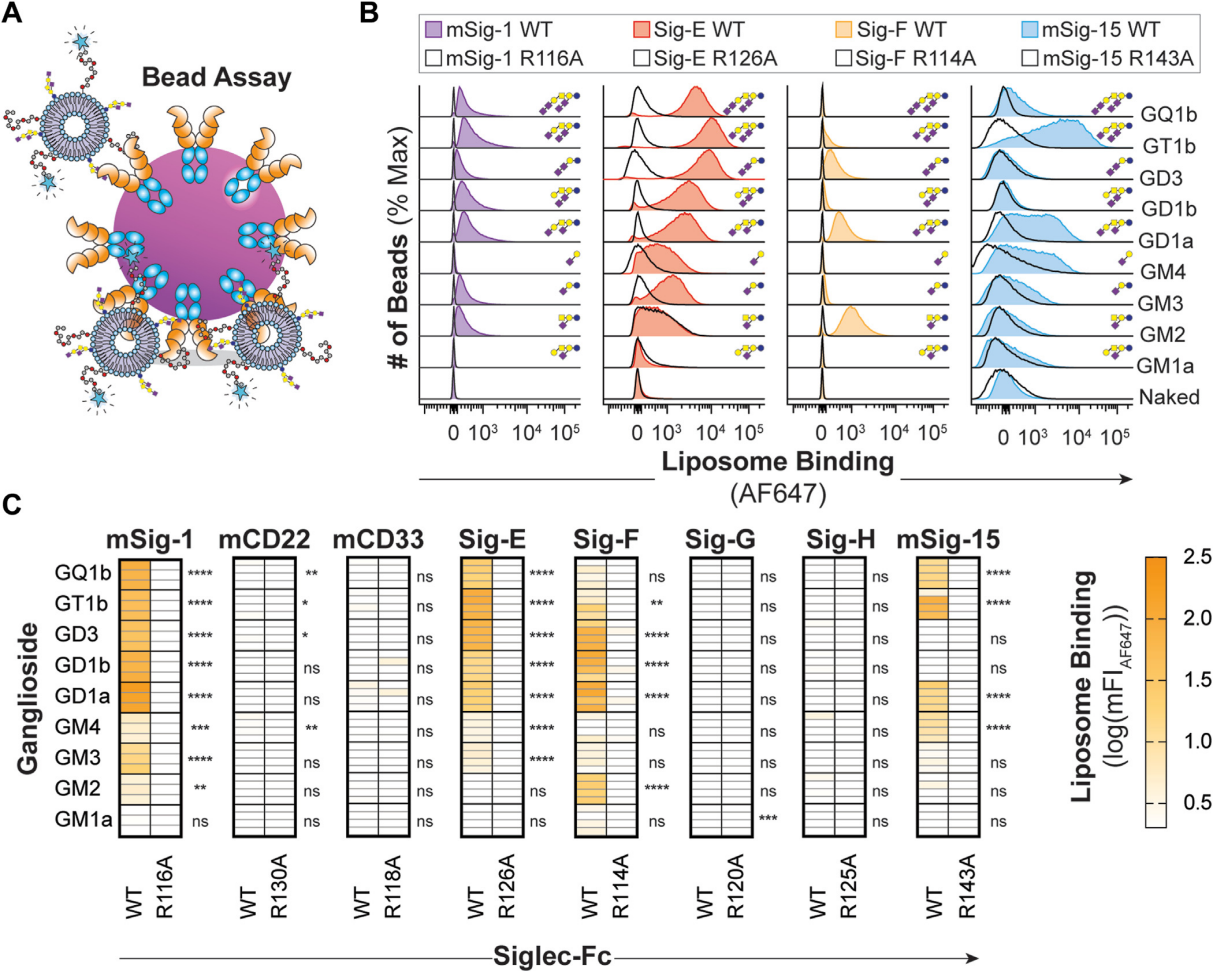
**Interrogation of mSiglecs against ganglioside liposomes.***A*, schematic representation of the bead assay where the Siglec-Fc, featuring a C-terminal Strep-tag II, is precomplexed with streptavidin-coated microbeads and gangliosides are embedded in fluorescently labeled liposomes and binding is read out *via* flow cytometry. *B*, representative flow cytometry histograms of mSiglec-1, Siglec-E, Siglec-F, mSiglec-15, and their corresponding arginine mutants binding to ganglioside liposomes. *C*, heatmaps summarizing ganglioside liposome binding to mSiglecs. Color is representative of the mean log_10_(mFI_AF647_) of each ganglioside liposome subtracted from the log_10_(mFI_AF647_) of the same ganglioside against the corresponding arginine mutant from at least three technical replicates. For (*C*), a one-way ANOVA was used to compare the binding between the WT Siglec and the corresponding arginine mutant. Not Significant (NS), *p* > 0.05; ∗0.05 > *p* ≥ 0.01; ∗∗0.01 > *p* ≥ 0.001; ∗∗∗0.001 > *p* ≥ 0.0001; ∗∗∗∗*p* < 0.0001.

To study the ability of mSiglecs to recognize glycolipids, we profiled the binding of Siglecs to a panel of nine gangliosides using our previously optimized liposome formulation, which consists of 3 mol% ganglioside, 0.5 mol% PEG_45_-DSPE, 58.5 mol% PSPC, and 38 mol% cholesterol (15). The binding between the mSiglecs and gangliosides was first studied using a bead assay wherein a Siglec-Fc bearing the Strep-tag II was immobilized on streptavidin microbeads, incubated with fluorescent liposomes containing gangliosides, and quantified by flow cytometry (Fig. 1*A*). mSiglec-1, Siglec-E, Siglec-F, and mSiglec-15 all bound multiple gangliosides, whereas no binding was observed between the other Siglecs and the ganglioside liposomes (Fig. 1, *B* and *C*). mSiglec-1, Siglec-E, and Siglec-F bound to nearly all gangliosides liposomes, albeit with different preferences. Surprisingly, none of these Siglecs engaged liposomes bearing GM1a. For mSiglec-1, GD1a bearing liposomes had the strongest binding, whereas liposomes formulated with GT1b had the strongest interaction with Siglec-E, and Siglec-F had the strongest binding to liposomes formulated with GM2. mSiglec-15 interacted preferentially with liposomes formulated with gangliosides, which possessed an α2-3 Sia on the terminal Gal, such as GM4, GT1b, and GD1a while also binding GQ1b.

### Investigating mSiglec binding to gangliosides outside a bilayer

**Figure 2:**
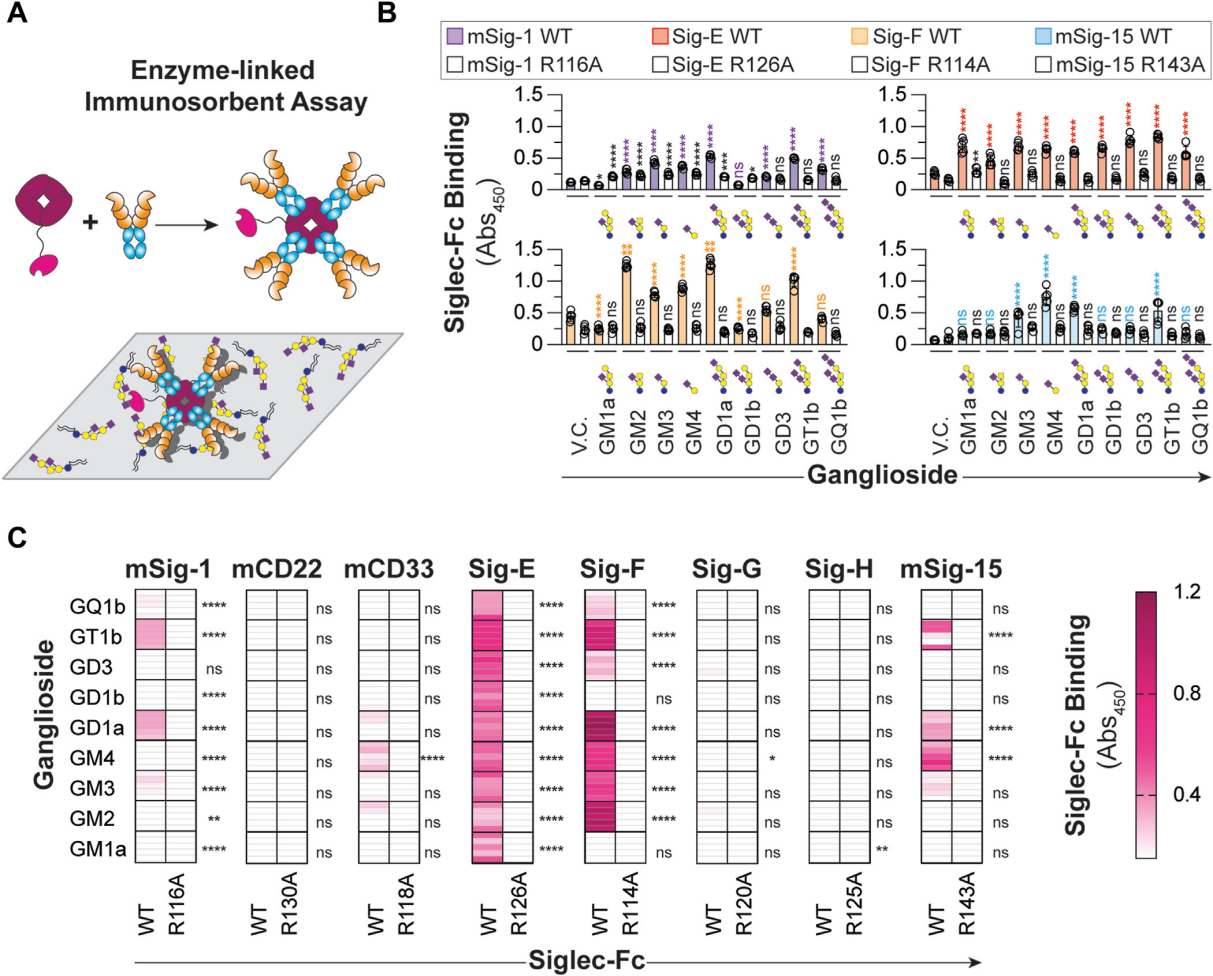
**Recognition of gangliosides by mSiglecs is affected by presentation.***A*, schematic representation of the ELISA where Siglec-Fc is precomplexed with Strep-Tactin-HRP and gangliosides are adsorbed to a microplate. *B*, representative ELISA results of mSiglec-1, Siglec-E, Siglec-F, mSiglec-15, and their corresponding arginine mutants binding to adsorbed gangliosides. *C*, heatmaps summarizing mSiglec-Fc binding to adsorbed gangliosides. Color is representative of the mean binding of the WT mSiglec-Fc complex to the adsorbed gangliosides liposome subtracted from the corresponding mutant mSiglec-Fc complex binding to the same ganglioside at least four technical replicates. For (*B*), a one-way ANOVA was used to compare the binding between the WT Siglec–ganglioside interaction and vehicle control well. For (*C*), a one-way ANOVA was used to compare the binding between the WT Siglec and the corresponding arginine mutant. Not Significant (NS), *p* > 0.05; ∗0.05 > *p* ≥ 0.01; ∗∗0.01 > *p* ≥ 0.001; ∗∗∗∗*p* < 0.0001. HRP, horseradish peroxidase.

As the oligosaccharide presentation of a glycolipid can affect its accessibility to a lectin (15, 34, 35), we assessed mSiglec-ganglioside binding through a traditional plate-based ELISA wherein the gangliosides are adsorbed to a microplate. The Siglec-Fc was precomplexed with Strep-Tactin horseradish peroxidase, incubated with the immobilized gangliosides in a well, washed, and colorimetrically developed (Fig. 2*A*). Similar to the bead assay, mSiglec-1, Siglec-E, Siglec-F, and mSiglec-15 bound multiple gangliosides (Fig. 2, *B* and *C*). Nevertheless, not every interaction from the bead assay was observed in the ELISA. For example, precomplexed mSiglec-1 engaged with GM3, GD1a, GT1b, and GQ1b in the ELISA whereas mSiglec-1-beads interacted with all gangliosides other than GM1a to some extent in the bead assay. Precomplex mSiglec-15 did not bind GQ1b in the ELISA, but mSiglec-15-beads did bind GQ1b in the bead assay. Conversely, some Siglec–ganglioside interactions were observed in the ELISA that were not observed in the bead assay. For instance, the precomplex Siglec-E engaged GM1a in the ELISA but not the bead assay. Overall, the results from the bead assay and the ELISA largely agree, demonstrating that mSiglec-1, Siglec-E, Siglec-F, and mSiglec-15 are proficient at interacting with gangliosides, but there are subtle differences in the ganglioside-binding profile between approaches and not all interactions are observed when the glycolipid is presented from a lipid bilayer.

### Re-optimization of liposome formulation does not reveal GM1a–mSiglec-1 interaction

**Figure 3:**
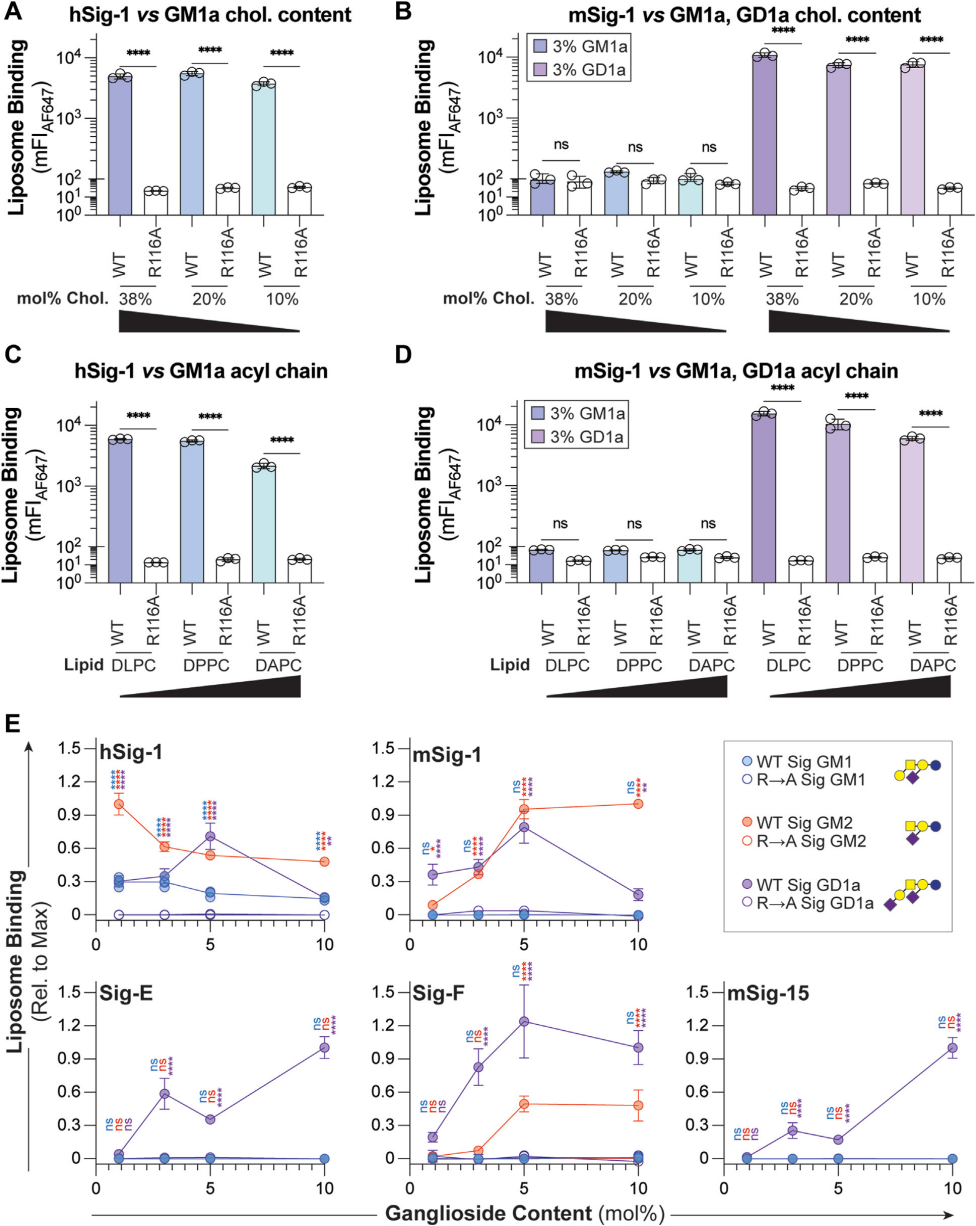
**Optimization of liposome formulation parameters for murine leclec-1.***A* and *B*, cholesterol content titration of GM1a and GD1a liposomes against h/mSiglec-1 respectively. *C* and *D*, acyl chain length titration of GM1a and GD1a liposomes against h/mSiglec-1, respectively. *E*, GM1a, GM2, and GD1a content titration (1–10 mol% of total lipids in the liposome) against hSiglec-1, mSiglec-1, Siglec-E, Siglec-F, and mSiglec-15, as well as their respective arginine mutants. Data is represented as the mean of at least three technical replicates, and error bars are representative of one SD from the mean. For panels (*A*–*D*), a one-way ANOVA was used to compare liposome binding between the WT and mutant Siglec at the same cholesterol content or acyl chain length. For (*E*), a one-way ANOVA was used to compare the binding between the WT Siglec and the corresponding arginine mutant at each mol% of ganglioside. Not Significant (NS), *p* > 0.05; ∗0.05 > *p* ≥ 0.01; ∗∗0.01 > *p* ≥ 0.001; ∗∗∗∗*p* < 0.0001. DLPC (12-carbon), DPPC (16-carbon), DAPC (20 carbon). *Blue*-GM1, *red*-GM2; *purple*-GD1a; Chol, cholesterol.

With the exception of Siglec-E in the ELISA, it was unexpected that no interactions between any mSiglec and GM1a were observed. The structural similarity between the oligosaccharide of GM1a and other gangliosides that did bind murine Siglecs motivated an investigation into why no murine Siglec interacted with GM1a–bearing liposomes. We posited that since our previous formulation was optimized against hSiglec-1 (15), it may not be optimal for murine Siglecs. Accordingly, we titrated the cholesterol content (38, 20, and 10 mol%) and the length of the acyl chain used for the bulk lipid in the liposome (12 carbon-DLPC, 16 carbon-DPPC, 20 carbon DAPC) in 3 mol% GM1a and GD1a liposomes against mSiglec-1 (Fig. 3, *A*–*D*). While both cholesterol content and acyl chain length had modest effects on Siglec engagement of ganglioside liposomes, no significant interaction between mSiglec-1 and GM1a-bearing liposomes was observed against any of the formulations tested. During the optimization of the liposome formulation in our previous work, ganglioside content (ligand density) was found to strongly influence Siglec–liposome interactions. To assess if the ganglioside content was responsible for the lack of GM1a binding to mSiglecs, we titrated the amount of three gangliosides (GM1a, GM2, and GD1a) from 1 to 10 mol% against the four mSiglecs that were found to engage with gangliosides using hSiglec-1 as a reference (Fig. 3*E*). In line with our previous study, hSiglec-1 bound GM1a, GM2, and GD1a, with GM2 being the best ligand (15). For the mSiglecs, GM1a did not to bind to mSiglec-1, Siglec-E, Siglec-F, or mSiglec-15 at any mol% tested, whereas GD1a was found to be a ligand for all the Siglecs tested. Like our initial screen of the mSiglecs (Fig. 1), GM2 was found to be a ligand for mSiglec-1 and Siglec-F and not for Siglec-E or mSiglec-15. However, there was a difference in the ligand density effects between mSiglec-1 and hSiglec-1 with GM2. These results suggest that the lack of engagement of GM1a by murine Siglecs is not due to an unoptimized liposomal formulation.

### mSiglec-1 prefers terminal α2-3–linked gangliosides

**Figure 4:**
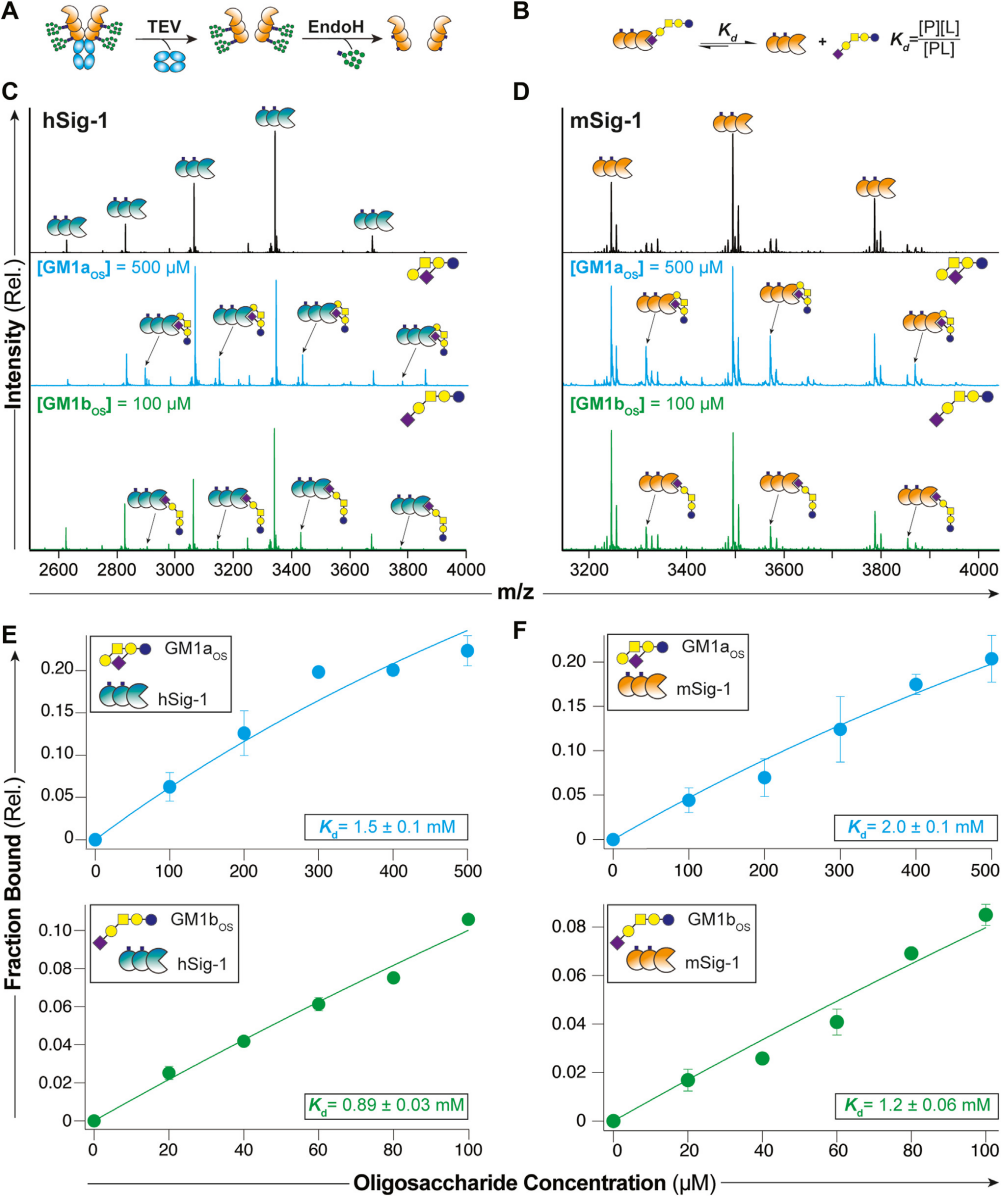
**Quantifying the interactions between m/hSiglec-1 and the oligosaccharide of GM1a and GM1b by a native mass spectrometry binding assay.***A*, schematic for the preparation of the Siglec-1 fragment from CHO Lec1 cells to eliminate heterogeneity from *N*-glycans. *B*, depiction of the equilibrium between Siglec-bound GM1b oligosaccharide and free oligosaccharide used to determine disassociation constants (*K*_d_). *C* and *D*, representative mass spectra for the binding between m/hSiglec-1 fragment to the oligosaccharide of GM1a (500 μM) and GM1b (100 μM). *E* and *F*, summary of ganglioside oligosaccharides titrations binding between m/hSiglec-1 fragment to the oligosaccharide of GM1a (100–500 μM) and GM1b (20–100 μM).

As mSiglec-1 engages liposomes bearing GD1a and GM2 but not GM1a and considering where the sialic acid(s) are presented from the oligosaccharide structures of GM1a, GM2, and GD1a, we hypothesized that the sialic acid presented from the internal Gal residue is difficult for mSiglec-1 to access. It follows that because GD1a contains Sia at both the internal and external Gal residues and was recognized by mSiglec-1, it suggests that recognition of GD1a by mSiglec-1 is through the terminal Gal residue. To understand the contribution to mSiglec-1 binding for each sialic acid residue on GD1a, we tested the ability of mSiglec-1 to bind GM1b, a linear structural isomer of GM1a where the sialic acid is linked to the terminal Gal instead of the internal Gal. The ability of h/mSiglec-1 to bind the oligosaccharides of GM1a and GM1b was tested using a quantitative native mass spectrometry–based assay developed previously to study Siglec–ligand interactions (15, 36, 37) (Fig. 4, *A* and *B*). In this way, the dissociation constants (*K*_*d*_) of hSiglec-1 and mSiglec-1 towards GM1a and GM1b were determined by measuring the relative abundance of monomeric Siglec in complex with the oligosaccharide compared to unbound Siglec (Fig. 4, *C* and *D*). The concentration of ganglioside oligosaccharide was then increased (100–500 μM form GM1a, 20–100 μM GM1b) and the change in the amount of Siglec–oligosaccharide complex was determined (Fig. 4, *E* and *F*). The interaction between hSiglec-1 and GM1b was found to be the strongest (*K*_d_ = 0.89 mM), followed by mSiglec-1 with GM1b (*K*_d_ = 1.2 mM), hSiglec-1 with GM1a (*K*_d_ = 1.5 mM), and the weakest interaction was between mSiglec-1 and GM1a (*K*_d_ = 2.0 mM). These results suggest that both mSiglec-1 and hSiglec-1 prefer terminal α2-3 sialic acids but that hSiglec-1 is better able to accommodate the internal α2-3–linked sialic acid in GM1a compared to mSiglec-1.

**Figure 5:**
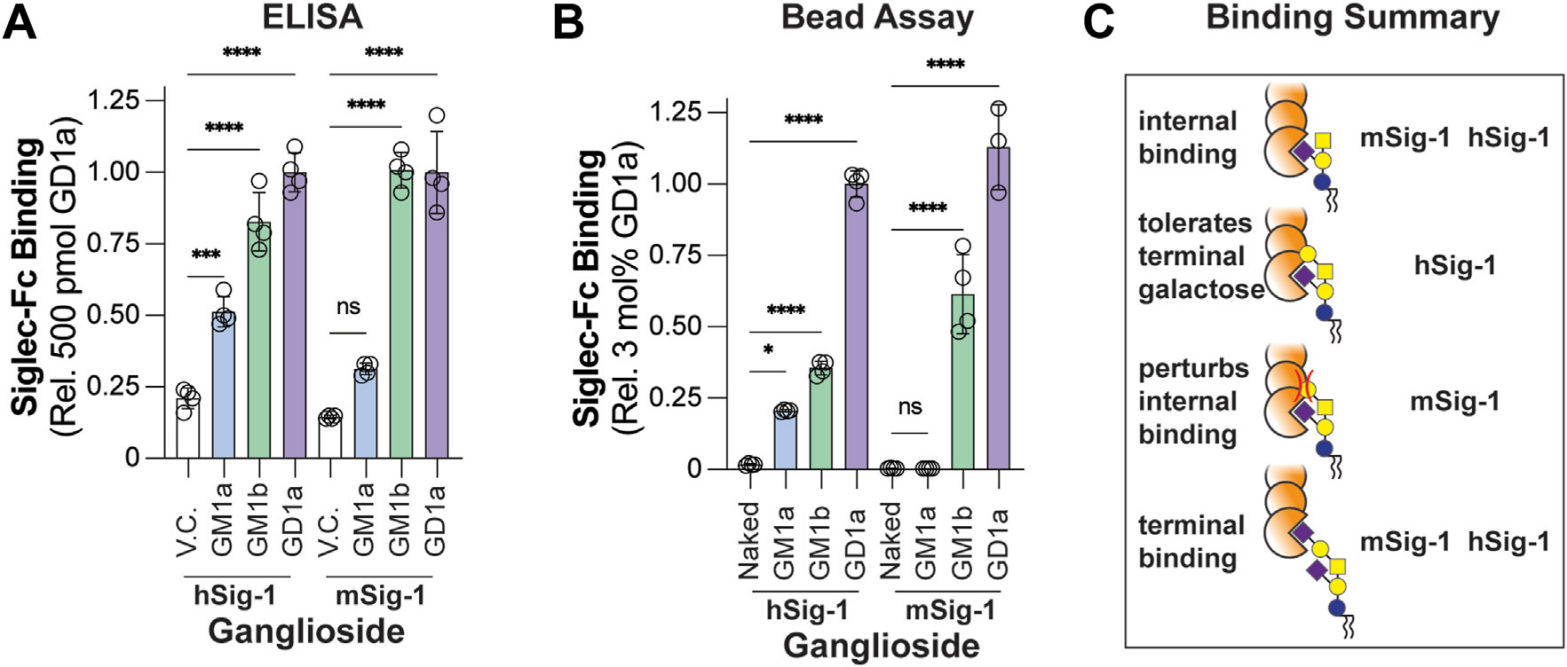
**Comparative binding of internal and externally linked sialic acids to m/hSiglec-1 in the bead assay and ELISA.***A* and *B*, hSiglec-1 and mSiglec-1 binding to GM1a, GM1b, and GD1a in the ELISA and bead assay, respectively. *C*, proposed model for human mSiglec-1 binding of gangliosides. Data is represented as the mean of at least three technical replicates and error bars are representative of one SD from the mean. For (*A*) and (*B*), a one-way ANOVA was used to compare the binding of each ganglioside to either a vehicle control (V.C.) or a naked liposome respectfully. Not Significant (NS); *p* > 0.05; ∗0.05 > *p* ≥ 0.01; ∗∗∗0.001 > *p* ≥ 0.0001; ∗∗∗∗*p* < 0.0001.

The results from the mass spectrometry assay, above, were followed up using the ELISA and bead assay. In the ELISA, hSiglec-1 showed the best binding to GD1a, followed by GM1b, and then GM1a. Recognition of GD1a and GM1b by mSiglec-1 was similar, but no significant binding to GM1a was observed (Fig. 5*A*). In the bead assay, GD1a was found to be a superior ligand than GM1b for both murine and human Siglec-1 and GM1a was found to be a ligand for hSiglec-1 but not mSiglec-1 (Fig. 5*B*). These results further support that mSiglec-1 poorly recognizes GM1a, particularly in the context of a lipid bilayer due to the sialic acid residue being linked to the internal Gal residue (Fig. 5*C*).

## Discussion

The cellular membrane of all mammalian cells contains a high density of glycolipids. Within the nervous system, the majority of sialic acid is presented from gangliosides (38). Given their ubiquitous nature, Siglec–ganglioside interactions are likely to play many important roles. For example, gangliosides presented from nanoparticles that occur naturally, such as viruses and extracellular vesicles, have the potential to be involved in a broad range of physiological and pathophysiological processes (15, 39, 40). Indeed, gangliosides within the membrane of HIV, Ebola, and SARS-CoV-2 are known to engage Siglecs and facilitate immune evasion and infection (31, 32, 33). *Campylobacter jejuni* uses ganglioside-like oligosaccharides, as a form of host mimicry, to engage Siglecs (41, 42). Moreover, cancerous cells release EVs with higher ganglioside content likely to protect themselves from the immune system (43). Many of these findings have been explored using *in vitro* approaches and few have been explored *in vivo*. Mice are the ideal model organism to pursue these findings, particularly because other model organisms—flies or worms—are not known to have Siglecs (9). Murine models have proven particularly useful in studying the roles of Siglecs in cancer and developing potential Siglec/sialic acid–based therapeutics (44, 45). Considering all these factors, murine models are well-suited for further elucidating the roles of Siglec–ganglioside interactions in humans. For these reasons, we were motivated to provide a deeper understanding of the similarities and differences between murine and human Siglec-ganglioside binding.

When comparing the ganglioside binding profiles between hSiglecs and mSiglecs, it is important to consider that all the gangliosides used in this study contained Neu5Ac as the form of sialic acid, and not Neu5Gc. Mice produce Neu5Gc in addition to Neu5Ac as their sialic acid and Neu5Gc-containing gangliosides could be preferentially recognized by some mSiglecs over their Neu5Ac-containing ganglioside counterparts (46, 47). While it would have been ideal to test Neu5Gc-containing gangliosides, unfortunately these are not readily available, and their synthesis is nontrivial. How Neu5Gc on gangliosides affects Siglec binding is an excellent future direction. Nevertheless, the use of Neu5Ac gangliosides enabled the direct comparison of the results from this work to our previous study where we analyzed binding of human Siglecs to gangliosides in similar types of assays.

**Figure 6:**
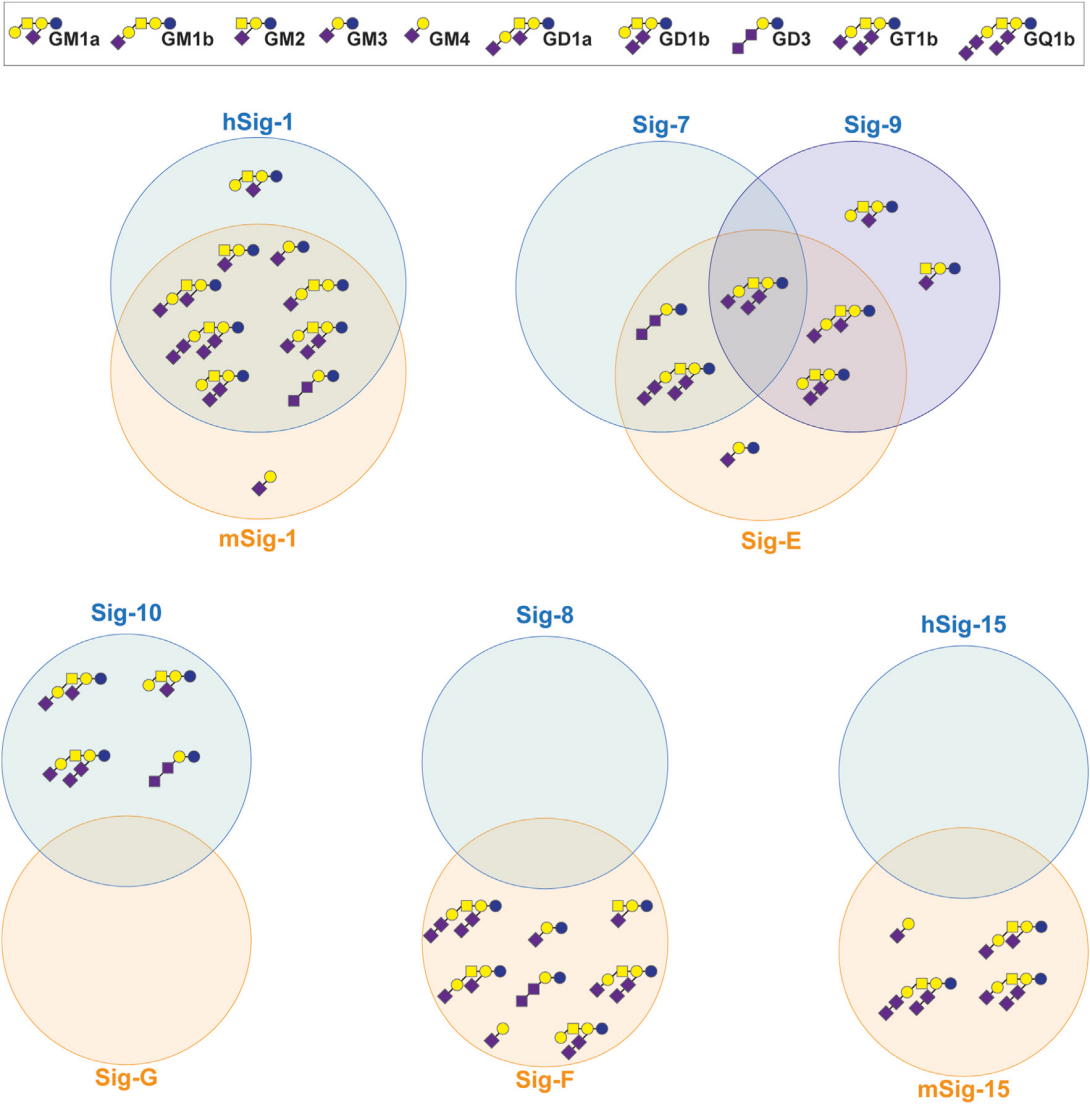
**Venn diagram comparison of murine and human Siglec ganglioside binding.** Interactions reported are a summary of interactions identified in this study and our precious study (15).

Comparing the results of the liposome-based assay from this study to those from our previous study (15), some of the mSiglecs bind more similarly to the analogous hSiglec than others when gangliosides are presented from a liposome (Fig. 6). For the conserved Siglecs, hSiglec-1 and mSiglec-1 bound all the same gangliosides with the exception of mSiglec-1 being unable to bind GM1a. The inability of mSiglec-1 to bind GM1a was previously reported using a cell adhesion assay (24). In this assay, which is similar to an ELISA, gangliosides were adsorbed to a microplate and then CV-1 in origin, and SV40 cells expressing full-length membrane-bound Siglecs were added to the plate. Unbound cells would then be washed away and the level of binding between the Siglec and the ganglioside was quantified by the activity of lactate dehydrogenase post cell lysis, which was proportional to the strength of the interaction between the Siglec and the ganglioside. Both m/hSiglec-1 are generally considered to recognize α2-3 sialic acid (8); however, the ability of both m/hSiglec-1 to bind α2-8–linked sialosides may be unappreciated, as both m/hSiglec-1 were found to bind α2-8 Sia gangliosides such as GD3, GD1b, and GQ1b (15). However, it is difficult to fully rule out that a portion of the α2-8–linked sialoside was hydrolyzed during the course of liposome preparation and led to these results. Consulting crystal structures of mSiglec-1, PDB 1OD7 (48) and 1QFO (49), the latter of which is co-crystalized with α2-3 sialyllactose (GM3 oligosaccharide), a co-crystal structure of m/hSiglec-1 with a α2-8 linked sialoside would be very insightful with respect to how Siglec-1 engages/accommodates the α2-8 sialic acid. In our previous study, we did not observe any binding of hSiglec-15 to gangliosides (15), yet mSiglec-15 did show ganglioside binding in this study. This is surprising given that m/hSiglec-15 share strong sequence homology (V-set identity 94%). While the reason(s) for the differences in ganglioside binding between h/mSiglec-15 are unclear, mSiglec-15 appeared to have a strong preference for a terminal α2-3–linked Neu5Ac as demonstrated by its ability to bind GM4, GD1a, and GT1b while not binding internally α2-3–linked gangliosides such as GM1a and GM2. This is in line with previous investigations that also observed Siglec-15 binding to α2-3–linked sialic acids (37, 50).

For the CD33-related Siglecs, while hCD33 and mCD33 share a name, the two Siglecs are structurally and functionally different (2). With respect to hCD33, ganglioside binding has only been observed using approaches where the ganglioside is outside a bilayer (17). In agreement with this, we observed only minimal binding between mCD33 and GM3 in the ELISA but not in the bead assay. For orthologs, Siglec-7, Siglec-9, and Siglec-E, all bound most gangliosides in this study and in other investigations (15, 26). Previously, Siglec-10 was found to bind many gangliosides (15, 51), whereas in this study, Siglec-G was unable to bind any gangliosides regardless of presentation. With respect to the paralogs Siglec-F and Siglec-8, Siglec-8 has not been reported to bind gangliosides when presented from a liposome (15) but can bind gangliosides outside a bilayer (26). In this study, Siglec-F could bind all the gangliosides that Siglec-8 was reported to bind to in an ELISA but could also bind GM4, GD1a, and GD1b when presented from a liposome. Both the bead assay and the ELISA are robust assays for measuring Siglec–ganglioside interactions. However, it is important to consider that these assays are artificial and due to the multivalency leveraged in both platforms, the observed interactions, and effects of ganglioside content on Siglec binding should be validated in a more *bona fide* biological membrane in the future.

GM1a is an important molecule in mammalian physiology and pathophysiology as it is one of the most common gangliosides (38, 52). In Huntington’s Disease murine models, delivering GM1a can restore healthy motor functions (53). With GM1a involved in many physiological and pathophysiological processes, it was surprising that none of the murine Siglecs bound GM1a in a bilayer. Even re-optimizing the liposome formulation with respect to cholesterol content, acyl chain length, and ganglioside content did not reveal any binding between mSiglec-1 and GM1a. However, these optimizations are not exhaustive and perhaps in the context of a bonafide biological membrane, mSiglec-1 may be able to interact with GM1a as binding between mSiglec-1 and GM1a could be observed in the mass spectrometry assay. However, the interaction between mSiglec-1 and GM1a was the weakest interaction measured. While mSiglec-1 does have a weaker affinity to GM1a than hSiglec-1, the difference is not large enough to explain the observed difference in the bead assay and the ELISA and is more likely due to other factors such as the oligosaccharide presentation.

In our focused ELISA, hSiglec-1 bound GD1a with greater avidity than GM1b whereas mSiglec-1 bound the two gangliosides equally. This may be because hSiglec-1 can bind to the internal and terminal Sia residues, whereas mSiglec-1 can only bind the terminal Sia residue. In the GM1-focused bead assay, both hSiglec-1 and mSiglec-1 bound GD1a with greater avidity than GM1b. This may be due to GM1b having a different optimal ganglioside content than GD1a, resulting in an unfavorable oligosaccharide conformation and decreased engagement of the Siglec. Alternatively, the branched nature of GD1a may position the terminal Sia towards the solvent allowing for engagement by Siglec-1, whereas the linear GM1b oligosaccharide may adopt a more buried or ‘laid down’ conformation proposed for other linear gangliosides like GM3 (54). In the future, techniques such as STD-NMR or cocrystal structures can be used to directly assess which sialic acid residues are recognized by Siglecs. Overall, it appears that Siglec–ganglioside binding profiles are reasonably conserved between human Siglecs and their equivalent murine Siglecs.

The foundation of our understanding of the physiological and pathophysiological roles of Siglecs are rooted in studies that discovered and described Siglec ligands. However, these discoveries may not always translate perfectly to human biology. While many Siglecs are conserved or may serve the same function at the organism or cellular level in humans and mice, subtle differences in structure may have profound biological effects that need to be considered. The results from this study suggest that murine models are generally appropriate to study human Siglec–ganglioside interactions at a global level but are less appropriate when studying specific interactions such as Siglec-1 and GM1a.

## Experimental procedures

### Siglec-Fc expression and purification

Previously stably transfected Chinese hamster ovary cells (37) we cultured for 1 week post confluency in DMEM-F12 (Gibco) supplemented with 5% (V/V) fetal bovine serum (FBS-Gibco) and penicillin (Gibco, 100 U/ml), and streptomycin (Gibco, 100 μg/ml) at 37 °C and 5% CO_2_. Media was then collected and filtered through a 0.2 μM filter and stored at 4 °C. The Siglec-Fc was then purified using an AKTA FPLC and a HisTrap Excel (GE) column and then a Strep-Tactin column (IBA) as described previously (15, 36, 37, 55). Purified protein was then dialyzed, concentrated, and then lyophilized. Lyophilized Siglec-Fc was stored at −20 °C.

### Siglec-Fc ELISA

The Siglec-ganglioside ELISA was described previously (15), but in short, gangliosides were dissolved in ethanol at a concentration of 10 μM and 50 μl of solution was transferred to a microplate resulting in 500 pmol of ganglioside per well. The microplate was left to dry overnight at room temperature. The following day, the microplate was blocked with 5% (m/V) bovine serum albumin/PBS pH 7.4 for 1 h at room temperature. While blocking, the Siglec-Fc complex was formed by adding Siglec-Fc (2 μg/ml) to Strep-Tactin-HRP (0.13 μg/ml-IBA) for at least 30 min at room temperature. The plate was then washed with PBS and the Siglec-Fc was then added to each well and the plate was incubated at room temperature for 2 h. The plate was then washed with PBS and developed using TMB substrate. One molar of phosphoric acid was then added and the absorbance at 450 nm was measured using a Molecular Devices SpectraMAX iD5.

### Liposome production and extrusion

Liposomes were produced according to our previously optimized liposome formulation and procedure (15). Structural lipids (DSPC, PSPC, DSPE-PEG, and Cholesterol Avanti) were suspended in chloroform and transferred to a glass test tube. A gentle stream of N_2_ gas was used to remove the chloroform. Once all the chloroform was removed, 100 μl of DMSO was added to the tube followed by the functional lipids (gangliosides-Matreya LLC, AF647-PEG-DSPE-made in house (56)), and the mixture was placed in a −80 °C freezer until frozen. The DMSO was then removed *via* lyophilization, and the dry lipids were stored at −80 °C. Lipids were then allowed to warm to room temperature and hydrated with PBS and were extruded using an avanti mini-extruder. Liposomes were then stored at 4 °C.

### Siglec-Fc bead assay

The Siglec bead assay was described previously (15), but in short, Pierce streptavidin-coated magnetic beads (Thermo Fisher Scientific) were blocked with 2% (m/V) bovine serum albumin/PBS on ice for 1 h. The beads were then added to 25 μg/ml Siglec-Fc solution and left to rest on ice for 1 h. Siglec-Fc–coated beads were then transferred to a 96-well round bottom microplate, and excess solution was removed using a plate magnet. The Siglec-Fc beads were then resuspended in 50 μM liposome solution and incubated at 37 °C for 30 min. The beads were then washed with PBS, and then liposome binding was accessed by flow cytometry.

### Flow cytometry

Flow cytometry measurements were collected on a 5-laser Fortessa X-20 (BD Bioscience). All the resulting data were analyzed using FlowJo (10.5.3) software (BD Biosciences, www.flowjo.com).

### Siglec preparation for direct binding assay

Monomeric Siglec fragment was prepared as described previously (15, 36, 37).

### Direct ESI-MS–binding assay

Direct binding was performed as described previously (15, 36, 37). GM1a and GM1b oligosaccharides were purchased from Biosynth.

### Statistical analysis

A one-way ANOVA was used. All statistical analysis was carried out using GraphPad Prism version 9 (graphpad.com).

### Data collection software

Flow cytometry data were collected with BD FACSDivaTM software (bdbiosciences.com) Version 8.0.1 and analyzed with FlowJo LLC. Version 10.5.3. ELISA data was collected using Molecular Devices Soft Max Pro 7.0.3.

## Data availability

The authors declare that all relevant data can be found in this document. For access to the raw data, please contact the corresponding author (M.S.M.) which is kept electronically and will be forwarded upon request.

## Conflict of interest

The authors declare that they have no conflicts of interest with the contents of this article.
